# Non-neotissue constituents as underestimated confounders in the assessment of tissue engineered constructs by near-infrared spectroscopy

**DOI:** 10.1016/j.mtbio.2023.100879

**Published:** 2023-11-28

**Authors:** Omar Anwar Elkadi, Florencia Abinzano, Ervin Nippolainen, Ona Bach González, Riccardo Levato, Jos Malda, Isaac O. Afara

**Affiliations:** a Department of Technical Physics, University of Eastern Finland, Kuopio, Finland; b Department of Orthopedics, University Medical Center Utrecht, Utrecht University, 3584 CX, Utrecht, the Netherlands; c Department of Clinical Sciences, Faculty of Veterinary Medicine, Utrecht University, 3584 CT, Utrecht, the Netherlands

**Keywords:** Tissue engineering, Near-infrared spectroscopy, Machine learning, Confounders

## Abstract

Non-destructive assessments are required for the quality control of tissue-engineered constructs and the optimization of the tissue culture process. Near-infrared (NIR) spectroscopy coupled with machine learning (ML) provides a promising approach for such assessment. However, due to its nonspecific nature, each spectrum incorporates information on both neotissue and non-neotissue constituents of the construct; the effect of these constituents on the NIR-based assessments of tissue-engineered constructs has been overlooked in previous studies. This study investigates the effect of scaffolds, growth factors, and buffers on NIR-based assessments of tissue-engineered constructs. To determine if these non-neotissue constituents have a measurable effect on the NIR spectra of the constructs that can introduce bias in their assessment, nine ML algorithms were evaluated in classifying the NIR spectra of engineered cartilage according to the scaffold used to prepare the constructs, the growth factors added to the culture media, and the buffers used for storing the constructs. The effect of controlling for these constituents was also evaluated using controlled and uncontrolled NIR-based ML models for predicting tissue maturity as an example of neotissue-related properties of interest. Samples used in this study were prepared using norbornene-modified hyaluronic acid scaffolds with or without the conjugation of an N-cadherin mimetic peptide. Selected samples were supplemented with transforming growth factor-beta1 or bone morphogenetic protein-9 growth factor. Some samples were frozen in cell lysis buffer, while the remaining samples were frozen in PBS until required for NIR analysis. The ML models for classifying the spectra of the constructs according to the four constituents exhibited high to fair performances, with F1 scores ranging from 0.9 to 0.52. Moreover, controlling for the four constituents significantly improved the performance of the models for predicting tissue maturity, with improvement in F1 scores ranging from 0.09 to 0.77. In conclusion, non-neotissue constituents have measurable effects on the NIR spectra of tissue-engineered constructs that can be detected by ML algorithms and introduce bias in the assessment of the constructs by NIR spectroscopy. Therefore, controlling for these constituents is necessary for reliable NIR-based assessments of tissue-engineered constructs.

## Graphical abstract

**Figure:**
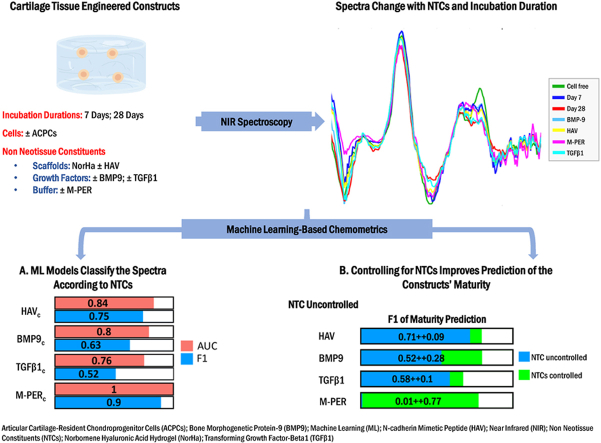

## Abbreviations

ACPCs: Articular Cartilage Resident Chondroprogenitor Cells
AUC: Area Under the Receiver Operating Characteristic Curve
BMP9: Bone Morphogenetic Protein-9
HAV: Histidine-Alanine-Valine
ML: Machine Learning
NIR: Near Infrared
NorHa: Norbornene-Modified Hyaluronic Acid
PBS: Phosphate Buffered Saline
SVM: Support Vector Machine
TECs: Tissue Engineered Constructs
TGFβ1: Transforming Growth Factor Beta1

## 1 Introduction

Tissue engineering is a promising regenerative medicine approach that aims to provide living functional constructs capable of restoring or improving damaged tissues. However, the assessment of tissue-engineered constructs (TECs) presents a challenge for the clinical translation of this approach [1]. Construct development is typically heterogeneous, with substantial differences existing between constructs cultured under the same conditions [2]. This heterogeneity undermines the reliability of the current gold standard methods (e.g., histology) for assessing the quality of TECs, which are destructive and thus require the analysis of samples that do not necessarily reflect the whole batch [[1], [2], [3]]. Moreover, these destructive methods require the sacrifice of the constructs, making them impractical for longitudinal monitoring of construct development during the expensive and time-consuming tissue engineering process [1,2]. This limitation generally restricts the number of samples that can be assessed, which are selected at limited time points, often at relatively long intervals (e.g., weekly intervals). This leads to the loss of temporal information that can be crucial for process optimization [1,2]. Therefore, there is a need for reliable non-destructive methods for assessing the quality of individual TECs before their clinical use, as well as to optimize the culture conditions.

Near-infrared (NIR) spectroscopy offers a promising approach for a rapid, non-destructive, and label-free assessment of TECs [1,2,[4], [5], [6], [7], [8]]. NIR spectra (800–2500 nm) reflect the chemical composition of the analyzed samples, mainly based on hydrogen-containing bonds (such as O–H, N–H, C–H, and S–H bonds), which are abundant in biological materials [1,2]. Due to the broad and overlapping bands in the NIR spectral range, NIR-based analysis of biological samples relies on the overall spectral pattern. These patterns act as a fingerprint for samples with specific properties, which can be used for tissue characterization via chemometrics based on multivariate analyses and machine learning (ML) [4,[8], [9], [10]]. With simple instrumentation and its non-destructive capability, NIR spectroscopy can also provide real-time information on neotissue-related biochemical and structural changes in the construct, making it suitable for *in situ* monitoring of tissue growth [1,2].

However, as a label-free method sensitive to nonspecific bonds, NIR spectra of TECs can also be affected by constituents in the samples that are not related to the neotissue. These non-neotissue constituents can be part of the construct (such as the extracellular matrix mimetic scaffolds), added during the culture (such as supplemented growth factors), or added to the samples post-culture (such as buffers used for storage and post-culture analyses). Some of these non-neotissue variables can comprise a significant proportion of the sample, which changes during tissue development. For instance, in the initial phases of cartilage tissue engineering, samples primarily comprise porous biodegradable scaffolds, which offer structural support for chondrogenic cells. The scaffold pores allow the influx of the culture media that provide nutrients and growth factors, facilitating cellular nourishment and chondrogenesis. Over time, the cells proliferate and produce their own extracellular matrix—mainly composed of collagens and proteoglycan*s. This matrix* gradually expands, replacing the degrading scaffold, and eventually becomes a substantial component of the mature cartilage constructs [2,[11], [12], [13], [14], [15]]. Thus, the spectra of constructs with varying non-neotissue constituents can be used to train and test ML models for assessing TECs. If these constituents also have a measurable effect on the NIR spectra of the constructs that can be detected by the employed ML algorithm, they can be potential confounders that can introduce bias into the model. It is important to identify and account for the effect of such confounders, as they can result in misleading conclusions that compromise the validity of the study and/or the performance of the models [16]. However, previous studies have overlooked the effect of these non-neotissue constituents on NIR-based assessments of TECs.

In this study, we investigated, for the first time, the effect of non-neotissue constituents on NIR-based assessments of TECs. First, to determine if these non-neotissue constituents have a measurable effect on the NIR spectra of the constructs, we evaluated the classification performance of nine ML algorithms in classifying the NIR spectra of cartilage TECs according to four non-neotissue constituents of the constructs independent of the maturity of the constructs. These four constituents were selected to represent the scaffolds used to prepare the constructs, the growth factors added to the culture media, and the buffers used in the preparation or storage of the samples. Then, we evaluated the effect of controlling for these constituents by comparing the performance of controlled and uncontrolled models for classifying the spectra of the constructs according to their maturity (as indicated by incubation duration) as an example of neotissue-related properties, which is the main objective of TEC assessment. We hypothesized that non-neotissue constituents induce measurable changes in the NIR spectra of TECs that can be detected by one of the commonly used ML algorithms. These changes make these constituents potential confounders that can introduce bias to models developed for the prediction of neotissue-related properties and can decrease the performance of the models if not controlled.

## 2 Materials and methods

### 2.1 Tissue culture

The samples analyzed in this study were prepared as part of a previous study that aims to assess the chondrogenic impact of an N-cadherin mimetic peptide (HAV) in combination with different growth factors (Unpublished). Articular cartilage-resident chondroprogenitor cells (ACPCs) were isolated from leftover cartilage tissue from total knee replacement surgeries following established protocols [17], and the ethical guidelines for “good use of redundant tissue for research” of the Dutch Federation of Medical Research Societies within the University Medical Center Utrecht [18]. The ACPCs were then expanded to passage 3 in chondroprogenitor expansion media, which were refreshed twice a week as described previously [17]. ACPCs (20 million/mL) were then encapsulated in two types of norbornene-modified hyaluronic acid hydrogels (NorHa). One type was further modified to carry an N-cadherin mimetic peptide sequence (**HAV**DIGGGC, ∼869.95 g/mol, GenScript), and the other type was used without further modifications. The gels were cast on 5 × 2 mm disks and crosslinked by exposure to visible blue light (405 nm) for 8 min at an intensity of ∼10 mW/cm^2^ in the presence of the photoinitiator lithium phenyl-2,4,6-trimethylbenzoylphosphinate (LAP, Sigma‒Aldrich, The Netherlands) and the crosslinker 11-dithiothreitol (DTT, Sigma‒Aldrich, The Netherlands).

The constructs were then incubated at a temperature of 37 °C, relative humidity of 95 % & 5 % CO_2_ in a chondrogenic medium composed of Dulbecco's modified Eagle medium (DMEM, 31,966, Gibco, The Netherlands), 1 % insulin-transferrin-selenous acid (ITS + Premix, Corning, The Netherlands), 0.2 mM ascorbic acid-2-phosphate (ASAP, Sigma, The Netherlands), 1 % penicillin and streptomycin (Life Technologies, The Netherlands) and 1 % 1 M HEPES (Gibco, The Netherlands). The culture media were supplemented with either 100 ng/mL bone morphogenetic protein-9 (BMP9) or 10 ng/mL transforming growth factor-beta1 (TGFβ1); the media were refreshed twice a week. The samples were harvested after 7 or 28 days. The harvested samples were washed with phosphate-buffered saline (PBS), and then frozen in 250 μl of PBS or Mammalian Protein Extraction Reagent (M-PER, Thermo Scientific, The Netherlands) until NIR analysis.

Control samples (n = 12) were prepared using the same procedures but without cells, growth factors, or HAV conjugation. Six of those control samples were incubated for 7 days, and the other six were incubated for 28 days. A summary of the contents of the test samples (n = 69) is listed in Table 1, while the details of the samples are listed in Supplementary Material 1.

**Table 1:** The distribution of the non-neotissue constituents in the tissue-engineered constructs incubated for 7 and 28 days.

Non-neotissue constituent		Day7 constructs (n = 41)	Day28 constructs (n = 28)	Total (n = 69)
**M-PER (%)**	No	30 (73.2)	18 (64.3)	48 (69.6)
	Yes	11 (26.8)	10 (35.7)	21 (30.4)
**HAV motif (%)**	No	17 (41.5)	12 (42.9)	29 (42.0)
	Yes	24 (58.5)	16 (57.1)	40 (57.9)
**BMP-9 (%)**	No	19 (46.3)	16 (57.1)	35 (50.7)
	Yes	22 (53.7)	12 (42.9)	34 (49.3)
**TGFβ1 (%)**	No	27 (65.9)	21 (75.0)	48 (69.6)
	Yes	14 (34.1)	7 (25.0)	21 (30.4)

Each sample may contain one or more of the non-neotissue constituents.

### 2.2 Near-infrared spectroscopy

The constructs were thawed at room temperature before spectroscopic measurement. NIR spectra (943.8–2491 nm) were acquired three times from each sample (a total of 240 spectra) using a system consisting of an NIR spectrometer (AvaSpec-NIR256-2.5-GSC, Avantes BV), a light source (Avalight-HAL-S, Avantes BV, The Netherlands) and a custom diffuse reflectance fiber optic probe. The probe tip window (*d* = 2 mm) contains 114 optical fibers (*d* = 100 μm), with 100 fibers emitting and 14 fibers collecting light to the spectrometer. During the measurements, the probe tip was placed perpendicular to and in contact with the center of the construct surface. The spectral acquisition was conducted using Avasoft software (version 8.7.0, Avantes BV), and the spectra were exported and then merged into a single table with appropriate labels for data analysis.

### 2.3 Data preprocessing and machine learning

**Fig. 1:**
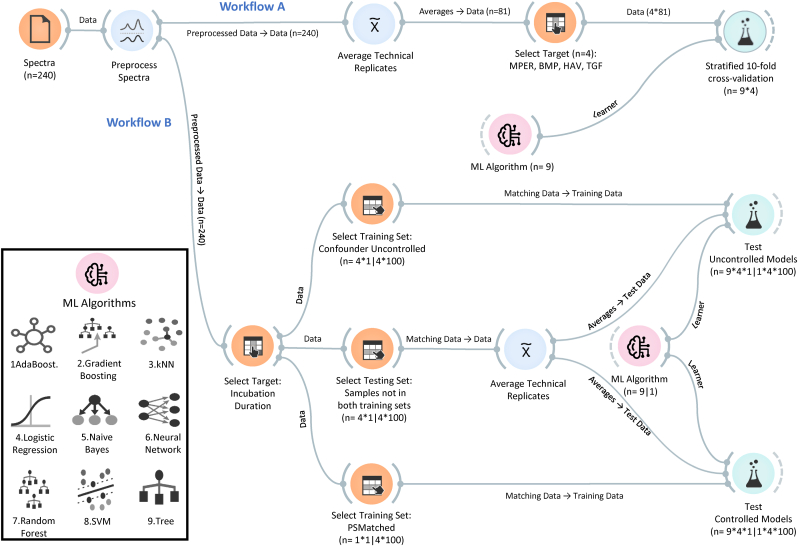
Overview of the machine learning workflows used in this study. Workflow A: The evaluation of the performances of nine machine learning (ML) algorithms by stratified 10-fold cross-validation in classifying the spectra according to the presence of M-PER reagent (MPER), the growth factors BMP-9 (BMP) and TGF-β1 (TGF), and HAV motif (HAV) in the constructs. Workflow B: the evaluation of the performances of a set of nine controlled models (propensity score matched: PSMatched) and corresponding uncontrolled models in classifying the spectra of the constructs according to their maturity as indicated by the incubation duration (7 days vs 28 days). The top-performing model was further evaluated by Monte Carlo cross-validation with random undersampling of Day 7 samples (100 iterations). The same workflow was used for each of the four confounders (non-neotissue constituents). The number of samples in the training and testing subsets in this workflow depends on the distribution of the tested confounder.

The spectra were imported into Orange 3.33 with the Quasar add-on (Bioinformatics Lab, University of Ljubljana, Slovenia) for spectral data preprocessing and primary ML analysis [19,20]. Further ML analyses were also conducted in RStudio [21]. An overview of the ML workflow is shown in Fig. 1, and the detailed workflow file is presented in Supplementary Material 2. Savitzky‒Golay smoothing coupled with first derivative preprocessing was applied to all spectra with a second-order polynomial and a window length of 13 before analyses [22].

#### 2.3.1 Classification of the constructs according to the non-neotissue constituents

The technical replicates of the 81 samples were averaged and then used to evaluate the performance of nine ML algorithms in classifying the spectra of the constructs according to the presence of each non-neotissue constituent. Default hyperparameters from Orange (Table 2) were used and stratified 10-fold cross-validation was used for the evaluation of the models (Workflow-A in Fig. 1).

**Table 2:** The nine machine learning algorithms used in the study and their hyperparameters.

Algorithm	Parameters
**AdaBoost**	Number of estimators = 50; Learning rate = 1; Classification algorithm: SAMME.R, Regression loss function: Linear.
**Gradient Boosting**	No of trees = 100, Learning rate = 0.1, Individual trees depth limit = 3, Smallest subsets size (stop splitting) = 2
**k-Nearest Neighbors**	No of neighbors = 7; Metric = Euclidean; Weight = Uniform
**Logistic Regression**	Regularization: Ridge (L2); C = 1
**Naive Bayes**	Non specified
**Neural Network**	Neurons in hidden layers = 100; Activation: ReLu; Solver: Adam; Maximal number of iterations = 200
**Random Forest**	Number of trees = 10; Smallest subset size (stop splitting) = 5
**Support Vector Machine**	Cost = 1; Regression Loss epsilon = 0.1; Kernel = RBF; Numerical tolerance: 0.001; Iteration limit = 100.
**Tree**	Maximum tree depth = 10; Smallest subsets size (stop splitting) = 5; Classification stops when majority reaches 95 %

#### 2.3.2 The effect of controlling for non-neotissue constituents on the classification of the constructs according to their maturity

The effect of controlling for the non-neotissue constituents was assessed by comparing the classification performance of controlled and uncontrolled ML models, based on the same ML algorithms, in classifying the spectra of the constructs according to their maturity as indicated by the incubation duration (7 days vs. 28 days). The workflow, depicted in Fig. 1 as Workflow B, involved two steps.

In the first step, the preprocessed spectra were divided into 5 training sets and 4 test sets. These sets were used to train and test a group of controlled models—using a propensity score-matched set—and 4 groups of uncontrolled models: MPER-Uncontrolled models using a training set with M-PER reagent uncontrolled; BMP-Uncontrolled models using a training set with BMP-9 uncontrolled; TGF-Uncontrolled models using a training set with TGFβ1 uncontrolled; and HAV-Uncontrolled models using a training set with HAV motif uncontrolled. For simplicity, the 37 spectra of the 12 control samples were excluded from both the training and test sets.

For the uncontrolled training sets, the Day-28 arm included all samples with the non-neotissue constituent to be uncontrolled (MPER, TGF, etc.), while they were excluded from the Day-7 arm. For instance, in the training subset of the MPER-Uncontrolled models, all Day-28 samples contained M-PER reagent (n = 30 spectra/10 samples; Table 1) and all Day-7 samples did not contain M-PER (n = 87 spectra/30 samples; Table 1).

For the controlled models, the confounders were controlled by propensity score matching using the MatchIt package in R, where the propensity scores were estimated with logistic regression, and the scores were used for nearest neighbor matching without replacement [23]. Each matched pair was then included together in the training subset (propensity score matched set). Samples that failed to obtain a match using propensity score matching (37 spectra/13 samples) were excluded from the training set of the controlled models (n = 60 spectra: D28 = 30|D7 = 30).

For each non-neotissue constituent, the test set included all samples that are not part of either its corresponding uncontrolled training subset or the training subset of the controlled set (the propensity score matched set). The number of spectra in the training and test subsets was selected based on the number of the Day-28 samples that contain the non-neotissue constituent, as Day-7 samples are more than Day-28 samples (Table 1). To avoid the replicate trap, we ensured that technical replicates were contained in the same subset (training or test subset), and they were averaged before testing in the test subsets [24].

For each of the nine ML algorithms described in section 2.3.1 (Table 2), the propensity score matched set was used to train the controlled models (n = 9; a model for each algorithm), and the other sets were used to train their corresponding models with the non-neotissue constituents uncontrolled (n = 9*4; a model for each algorithm for each non-neotissue constituent). For each non-neotissue constituent, the controlled and uncontrolled models were evaluated on the same corresponding test set (based on the non-neotissue constituent). The classification performance of each controlled ML model was compared to that of its corresponding uncontrolled model developed by the same ML algorithm. The differences in the performance of the algorithms between the controlled and uncontrolled models were analyzed to assess the confounding effect of the non-neotissue constituents on the prediction of the maturity of the constructs.

In the second step, the top-performing algorithm from the first step was evaluated by modified Monte Carlo cross-validation to further assess the confounding effect of the non-neotissue constituents on the performance of the models while addressing the potential bias introduced by the class imbalance and/or sample size differences. The preprocessed spectra were imported into Rstudio, and then the performances of controlled and uncontrolled support vector machine (SVM)-based models (using the same hyperparameters used in Orange; Table 2) were evaluated using 100 random combinations of training and test sets as described in the first step but with random undersampling of the Day-7 samples without replacement. For each combination, Day-7 samples were randomly selected (using random seeds) to match the number of Day-28 samples in the training set of the uncontrolled models to balance the number of Day-7 and Day-28 samples. Propensity scores-matched pairs of Day-7 and Day-28 samples were randomly selected in the propensity score-matched (Controlled) training set to match the number of samples in the uncontrolled training set. Samples that were not selected in both training sets (Controlled and Uncontrolled) were used in the test set for this combination. Before analysis, the full dataset was cleaned by dropping the wavelengths with no variance and correcting for samples with missing technical replicates (2 samples) by simple replacement. The SVM was performed using the Caret package in R, while the hyperSpec package was used for handling the spectral data [25,26].

#### 2.3.3 Classification performance metrics and statistical analyses

The performance of the ML models was assessed using the area under the receiver operating characteristic curve (AUC), and the F1 score (F1). For each non-neotissue constituent, the significance of the differences between the F1 and AUC scores of the 9 uncontrolled ML models and their corresponding controlled models (based on each algorithm) was tested with the Wilcoxon rank sum test. For the Monte Carlo cross-validation, a paired *t*-test was used to test the significance of the differences between the F1 scores of the 100 uncontrolled SVM models and their corresponding controlled SVM models. All statistical analyses were performed using the R Package ‘stats’ version 4.2.1 [27]; p-values are presented as two-sided.

## 3 Results

### 3.1 Classification of the constructs according to the non-neotissue constituents

**Fig. 2:**
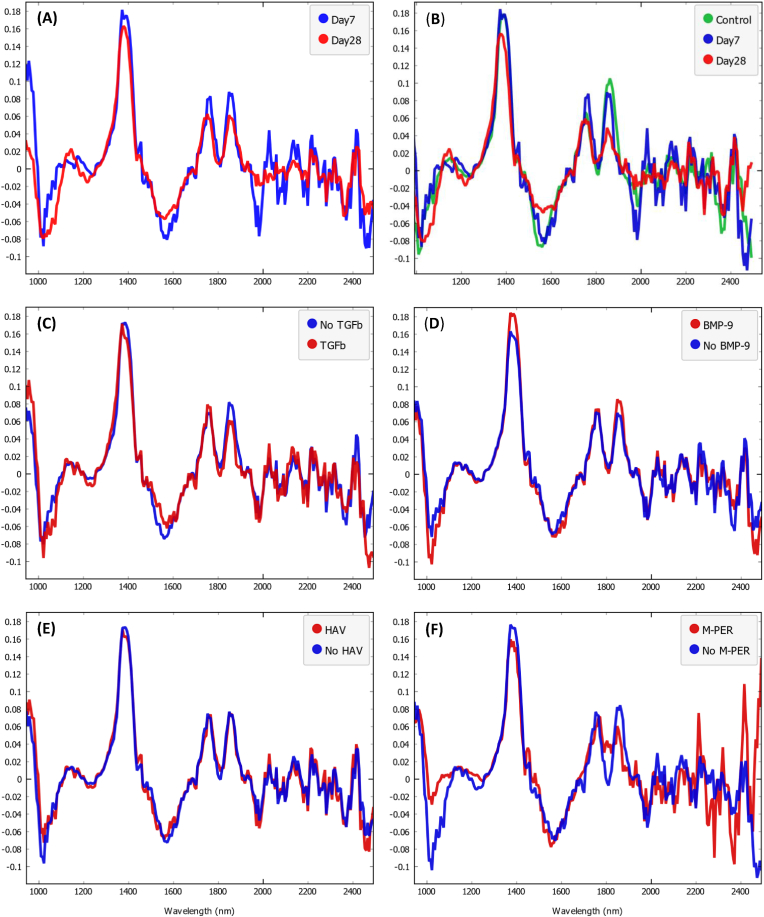
Mean preprocessed spectra (1st derivative) of the samples grouped according to the incubation duration (A), the incubation duration with controls as an independent group (B), and the presence of TGFβ1 (C), BMP9 (D), HAV motif (E), and M-PER reagent (F).

The mean preprocessed spectra of the samples (Fig. 2) show changes associated with each of the tested non-neotissue constituents. The spectral changes are more obvious with M-PER (Fig. 2F), but changes along the whole wavelength range can also be observed with HAV, BMP-9, and TGF-β1 (Fig. 2E, D, and C, respectively). At least one ML classifier with satisfactory F1 and AUC scores was developed for classifying the spectra of the constructs according to the presence of each of the four non-neotissue constituents. MPER-based classifiers exhibited the highest performance, followed by the classifiers based on the N- cadherin mimetic peptide HAV, then BMP-9, and finally TGF-β1 (Table 3).

**Table 3:** Selected performance metrics of the machine learning algorithms in classifying the constructs according to the non-neotissue constituents evaluated by stratified 10-fold cross-validation [AUC: Area under the Receiver Operating Characteristic curve].

Algorithm	M-PER		BMP-9		HAV		TGF-β1	
	AUC	F1	AUC	F1	AUC	F1	AUC	F1
AdaBoost	0.800	0.700	0.605	0.537	0.560	0.591	0.650	0.476
Gradient Boosting	0.889	0.737	0.807	0.635	0.768	0.675	0.758	0.522
kNN	0.958	0.769	0.671	0.414	0.694	0.597	0.739	0.414
Logistic Regression	0.922	0.667	0.618	0.213	0.586	0.538	0.572	0.000
Naive Bayes	0.919	0.632	0.626	0.619	0.650	0.525	0.708	0.531
Neural Network	1.000	0.900	0.770	0.636	0.838	0.747	0.781	0.439
Random Forest	0.983	0.865	0.648	0.588	0.640	0.588	0.808	0.424
SVM	0.992	0.900	0.713	0.519	0.778	0.675	0.761	0.083
Tree	0.817	0.683	0.592	0.493	0.589	0.595	0.533	0.429

**Fig. 3:**
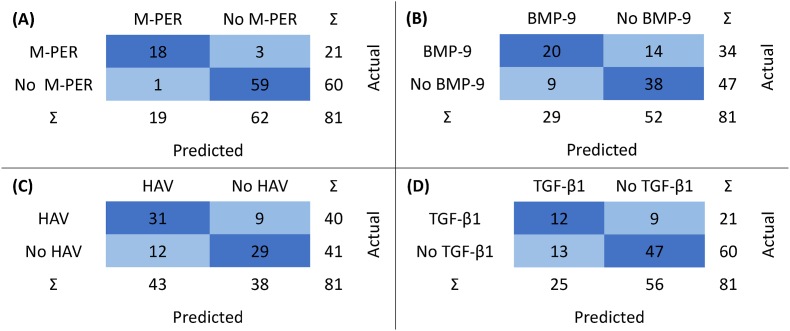
Confusion matrices of the top-performing models for the classification of the spectra of the constructs according to the presence of M-PER (A), BMP-9 (B), HAV motif (C), and *TGF-β1* (D). The top-performing models for M-PER (A) and the HAV motif (C) are based on neural network, while those for BMP-9 (B) and *TGF-β1* (D) are based on gradient boosting.

The classification of constructs according to the presence of M-PER reagent showed high AUC values ranging from 1 to 0.8 and F1 scores ranging from 0.9 to 0.632 (Table 3). The top-performing MPER classifier was based on the neural network algorithm and had 3 false negatives and 1 false positive (Fig. 3A), with an AUC of 1 and F1 of 0.9 (Table 3). The classification of constructs according to the presence of the HAV motif showed high to fair AUC and F1 values ranging from 0.838 to 0.56 and 0.747 to 0.525, respectively (Table 3). The top-performing HAV classifier was based on the neural network algorithm, which had 9 false negatives out of 40 positives and 12 false positives out of 41 negatives (Fig. 3C), with an AUC of 0.838 and an F1 of 0.747 (Table 3). The classification of constructs according to the presence of BMP-9 showed high to fair AUC values ranging from 0.807 to 0.592 and moderate to low F1 scores ranging from 0.635 to 0.493 (Table 3). The top-performing BMP9 classifier was based on the gradient boosting algorithm, which falsely predicted 14 samples as negatives (out of 34 positives) and falsely predicted 9 samples as positives (out of 47 negatives) (Fig. 3). This model exhibited an AUC of 0.807 and an F1 of 0.635 (Table 3). Similarly, the classification of constructs according to the presence of TGF-β1 exhibited high to fair AUC values ranging from 0.808 to 0.533 and fair to very low F1 values ranging from 0.531 to 0 (Table 3). The top-performing TGF-β1 classifier is based on the gradient boosting algorithm, which had 9 false negative predictions out of 21 positives and 13 false positive predictions out of 60 negatives (Fig. 3). This model had an AUC of 0.758 and an F1 of 0.522 (Table 3).

### 3.2 The effect of controlling for non-neotissue constituents on the classification of the constructs according to their maturity

The mean preprocessed spectra of the mature constructs (incubated for 28 days) show obvious differences compared to the immature constructs (incubated for 7 days), where the spectra of the immature constructs were closer to those of the controls (Fig. 2 A & B). Generally, the Uncontrolled ML models based on the 9 ML algorithms showed low performance in classifying the spectra of the constructs according to their maturity. However, after controlling for the non-neotissue constituents, significant improvement in the performance of the algorithms (in terms of both F1 and AUC scores) was observed in comparison to the uncontrolled models, except for the HAV-uncontrolled model, where the improvement in the AUC was marginal and not statistically significant (Table 4). The improvements obtained by controlling for MPER are the most significant, where most uncontrolled models failed to correctly classify any of the mature samples (Day 28 samples). Controlling for BMP9 resulted in an average percent increase of 23.6 % (P = 0.01277) and 23.5 % (P = 0.02427) in the F1 and AUC scores of the models based on the 9 algorithms, respectively. In contrast, controlling for HAV resulted in marginal improvement in the models' F1 and AUC values, with average percent increases of 14.4 % (P = 0.02071) and 1.8 % (P = 0.6236), respectively. Consistent with MPER and BMP9, controlling for TGFβ1 resulted in an average percent increase in the models’ F1 and AUC of 91.6 % (P < 0.0091) and 23.9 % (P = 0.01277), respectively (Table 4).

**Table 4:** F1 and AUC scores of the uncontrolled and controlled models in classifying the spectra of the constructs in their corresponding test sets according to their maturity.

Algorithm	MPER		BMP-9		HAV		TGF-β1	
	Controlled	Uncontrolled	Controlled	Uncontrolled	Controlled	Uncontrolled	Controlled	Uncontrolled
**F1 Scores**								
AdaBoost	0.880	0.143	0.556	0.533	0.750	0.632	0.667	0.316
Gradient Boosting	0.917	0.143	0.556	0.533	0.750	0.600	0.696	0.300
kNN	0.857	0.118	0.909	0.571	0.900	0.750	0.875	0.526
Logistic Regression	0.846	0.000	0.667	0.571	0.750	0.667	0.667	0.353
Naive Bayes	0.909	0.000	0.625	0.571	0.714	0.714	0.667	0.571
Neural Network	0.917	0.000	0.737	0.533	0.750	0.571	0.800	0.421
Random Forest	0.870	0.000	0.778	0.571	0.625	0.667	0.769	0.476
SVM	0.917	0.000	0.778	0.533	0.750	0.615	0.800	0.353
Tree	0.917	0.133	0.526	0.533	0.750	0.706	0.696	0.300
F1 Mean Difference (P Value)	0.832 (0.009091)		0.131 (0.01277)		0.0908 (0.02071)		0.335 (0.009091)	
**AUC**								
AdaBoost	0.833	0.479	0.667	0.672	0.804	0.716	0.686	0.507
Gradient Boosting	0.885	0.474	0.667	0.622	0.804	0.778	0.693	0.489
kNN	0.792	0.318	0.950	0.828	0.935	0.967	0.750	0.761
Logistic Regression	0.906	0.302	0.900	0.561	0.941	0.948	0.786	0.764
Naive Bayes	0.938	0.365	0.956	0.728	0.967	0.974	0.864	0.786
Neural Network	0.969	0.104	0.956	0.772	0.967	0.967	0.950	0.729
Random Forest	0.927	0.417	0.917	0.617	0.882	0.931	0.896	0.725
SVM	0.990	0.281	0.961	0.733	0.980	0.922	0.950	0.779
Tree	0.885	0.417	0.639	0.672	0.804	0.775	0.693	0.457
AUC Mean Difference (P Value)	0.552 (0.003906)		0.156 (0.02427)		0.01198 (0.6236)		0.141 (0.01277)	

**Fig. 4:**
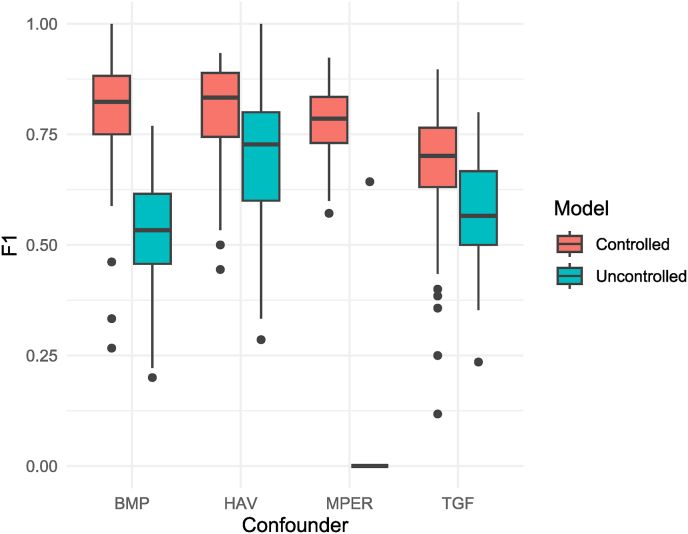
The F1 scores of the Monte Carlo cross-validation's 100 controlled and uncontrolled support vector machine models for classifying the spectra of the constructs according to their maturity showing the improvement in the performance of the models by controlling for each of the four confounders (non-neotissue constituents): BMP-9, HAV motif, M-PER reagent, and TGFβ1. All p-values are <0.0001.

Monte Carlo cross-validation of the support vector machine models also showed the significance of this improvement after controlling for each non-neotissue constituent (Fig. 4). Consistent with the performances of the primary ML models from the first step of the comparison, controlling for M-PER showed the most significant improvement in the F1 score, with a mean improvement of 0.77 (CI95: 0.75–0.79), where only a single model of the 100 uncontrolled models was able to correctly predict any of the day 28 samples. Controlling for BMP-9, TGF-β1, and HAV showed mean improvements of 0.28 (CI95: 0.25–0.30), 0.102 (CI95: 0.06–0.14), and 0.09 (CI95: 0.07–0.12), respectively.

## 4 Discussion

In this study, we demonstrated, for the first time, that non-neotissue constituents can be confounders that introduce bias in the assessments of TECs by NIR-based models. We demonstrated this by developing ML models for the classification of the NIR spectra of cartilage TECs according to four non-neotissue constituents with high to fair performances. We further supported this finding by showing that controlling for these four constituents significantly improves the performance of NIR-based models for the classification of the constructs according to their maturity—as indicated by their incubation duration. Generally, a confounder is a variable that affects both the independent and dependent variables, i.e., in terms of ML, a variable that affects both the input and output of the model [16]. In the context of this study, these non-neotissue constituents were identified as confounders due to their variability among the constructs, affecting the spectra of the constructs, and the ML algorithms can detect this effect. If left uncontrolled, this variability can compromise the prediction performance and reliability of the models [16,28].

The four non-neotissue constituents addressed in this study represent three important non-neotissue variables of a typical TEC sample: scaffolds, growth factors, and buffers. To represent the impact of practically relevant small changes in the scaffold, the constructs employed in this study were prepared using the hydrogel NorHA with or without the conjugation of the histidine-alanine-valine (HAV) motif in the first extracellular domain of N-cadherin as an N-cadherin mimetic peptide [29]. To represent the impact of changing the growth factors, the samples analyzed were incubated in media without growth factors or media supplemented with BMP9 or TGFβ1. BMP9 and TGFβ1 are commonly used growth factors in cartilage tissue engineering [30]. To represent the impact of buffers, samples were stored in PBS or M-PER reagent, which is a lysis buffer used to prepare samples for intracellular protein quantification assays [31].

The performance of the non-neotissue constituents-based classifiers varied from high, in the prediction of MPER, to fair, in the prediction of TGFβ1, with AUC and F1 scores ranging from 1 to 0.758 and 0.9 to 0.52, respectively. AUC and F1 scores were used as performance indicators because they are insensitive to class imbalance [32,33]. The high performance of the MPER classifier indicates a substantial change in the spectra associated with the presence of the buffer. This change is evident in the differences between the mean pre-processed spectra of the samples with and without M-PER (Fig. 2F), particularly between 1000 and 1100 nm. This substantial change in the spectra may be directly attributed to the M-PER reagent itself, owing to its high content in the samples, or indirectly due to its effect on the samples. MPER reagent contains a mild, non-denaturing detergent that solubilizes and extracts cellular total protein by dissolving cell membranes, preparing the samples for intracellular protein quantification assays [31]. Therefore, the addition of such reagents can significantly affect the chemical composition of the samples, invariably affecting their spectra. On the other hand, although only slight differences can be observed between the mean pre-processed spectra of the samples with and without the HAV motif (Fig. 2E), all models were able to detect subtle changes in the spectra associated with the presence of HAV. These changes may be attributed to the conjugated HAV motif or due to the processing effects on the NorHA gel during the conjugation of the HAV motif [34].

Similarly, the performances of the BMP9 and TGFβ1 classifiers reflect their ability to detect changes in the spectra of the samples (Fig. 2C and D) associated with the presence of these growth factors. Growth factors are typically used at low concentrations in cartilage tissue engineering similar to the concentrations used in this study [7,35], which can misleadingly undermine their capacity to affect the spectra of the constructs. However, despite their low concentrations, these growth factors can still cause detectable changes in the spectra of the constructs. These changes in the spectra can arguably be attributed to the optical response of these growth factors in the samples. For instance, the growth factors could be retained within the scaffold after each media change, gradually accumulating until their optical response reaches the limit of detection (the first measurement was after seven days of incubation, which included three media changes). In this case, the relatively low performance of the TGFβ1 classifiers can be attributed to its lower concentration in the samples. Alternatively, these changes could be attributed to the biological effect of the growth factors on the cells. For instance, BMP-9 and TGFβ1 bind to different receptors, which results in distinct downstream signaling pathways that can eventually lead to growth factor-dependent differences in the neotissue [30]. As the cells are abundant in the samples, these differences could be significant enough to affect the spectra, especially considering that the first measurements were collected after seven days of incubation. However, the detection of the growth factors by these classifiers is not related to the maturation of the constructs as they were able to identify the growth factors in both immature and mature constructs.

To assess the confounding effect of the four non-neotissue constituents, controlled models for classifying the spectra of the constructs according to their maturity were compared with corresponding uncontrolled models (with non-neotissue constituent uncontrolled) based on the same ML algorithms and evaluated on the same test sets. TEC maturity is a neotissue-related property of interest that was successfully estimated using NIR spectroscopy in previous studies, where the NIR spectra of the constructs were shown to be sensitive to the growth of the neocartilage [2,5,7,36]. The incubation duration was used as an indicator of the maturity of the constructs, where constructs incubated for 7 days were considered premature, while those incubated for 28 days were considered mature. After 28 days of culture, the constructs were expected to be significantly different from those cultured for 7 days due to the production of extracellular matrix components [7,37]. Yousefi et al. demonstrated that changes in the NIR spectra associated with the extracellular matrix production plateau after 28 days, with a corresponding plateau of the biochemical and mechanical properties of the constructs [7].

Consistent with the results of previous studies, the spectra of the mature constructs in the present study show obvious changes compared to the immature constructs, which in turn are closer to the spectra of the cells-free scaffolds (Fig. 2 A & B). This can be attributed to changes in the chemical composition of the constructs as a function of culture duration. The composition of the construct is dominated by the non-neotissue constituents (particularly the scaffold) in the early stages of the culture and then changes gradually due to the formation of the extracellular matrix during chondrogenesis [2,5,7,36].

However, despite these obvious differences, most uncontrolled models showed low performance and most of them exhibited lower performance when compared to the controlled model on the same testing set (Table 4). This low performance of the uncontrolled models can be attributed to the bias introduced by the non-neotissue constituents during model training. As all the instances of the mature constructs employed in training the uncontrolled models also include the uncontrolled non-neotissue constituent, the uncontrolled models were trained to identify the spectra of the mature constructs based on the patterns associated with the presence of non-neotissue constituents in addition to those associated with the maturation of the constructs. This likely confused the uncontrolled models when challenged with the test sets, as they were unable to detect patterns associated with the uncontrolled non-neotissue constituent in the spectra of the mature constructs. In the case of a non-neotissue constituent associated with strong changes in the spectra, such as M-PER, the model may lean towards changes induced by this constituent rather than the construct maturity, which explains the very low performance of the MPER-uncontrolled models, where most algorithms have F1 values of 0 (Table 4).

On the other hand, the controlled model was able to classify the spectra of the constructs according to their maturity (incubation duration) with high F1 and AUC (Table 4), even in the presence of M-PER despite its strong influence on the spectra. In this model, the non-neotissue constituents were controlled using propensity score matching, which is one of the common methods for controlling for multiple confounders. In propensity score matching, samples that have close propensity scores in each of the studied groups (immature and mature constructs in this study)—i.e., have more confounders in common—are paired together and then included together in the training subset to ensure that the confounders are controlled [28]. Thus, the controlled models were trained on datasets where the non-neotissue constituents are distributed equally between the instances of the immature and mature constructs. This enables the controlled models to exclude the patterns associated with the non-neotissue constituents, thus, minimizing their associated bias. The difference between the performance of the controlled and uncontrolled models reflects the confounding effect of these non-neotissue constituents on the assessment of construct maturity via NIR spectroscopy.

The approach used in the first step of the comparison between the controlled and uncontrolled maturity prediction models had two main limitations. First, the single partitioning of the data into training and test sets for each non-neotissue constituent can introduce bias attributed to this specific partitioning, where the selected samples may offer an advantage to one model over another. Second, although both the uncontrolled and the controlled models were tested on the same testing set, there is a possibility that the class imbalance in the training sets of some of the uncontrolled models (such as the models with TGFβ1 uncontrolled) can give an advantage to the controlled model. These limitations necessitated an additional approach in a second step.

To address these limitations, the algorithms that produced the top-performing models in the first step of the comparison were further evaluated using Monte Carlo cross-validation in the second step. In this approach, 100 controlled and uncontrolled SVM models were developed and evaluated by 100 random train-test splits. These 100 random combinations provided a more robust assessment of the models’ performance by reducing bias introduced by the specific partitioning of the data into training and test sets. The training sets for controlled models were also undersampled to match those of the uncontrolled models to avoid bias that might be introduced by class imbalance and the difference in the size of the training sets. The results of this more conservative approach are consistent with the results of the primary ML approach from the first step of the comparison and confirmed the improvement of the performance of the models by controlling for the non-neotissue constituents (Fig. 4). However, the model performances were generally lower than those of the primary ML models from the first step of the comparison, which could be attributed to the lower sample size resulting from undersampling [38].

Another limitation of this study is that we did not determine the fundamental changes within the spectra responsible for the classification and the exact cause of these changes. This is commonly accomplished by determining the exact wavelength regions that contribute to the models via feature importance analysis or by examining the model coefficients (for linear models) [10]. However, feature importance analysis cannot be performed in some algorithms, such as nonlinear SVM [39], which was the top-performing algorithm in predicting the maturity of the constructs in this study. Even if we determine the exact wavelength regions that contribute to the models, it will still be challenging to directly assign them to a specific component of the constructs due to the complexity of the NIR spectra with their broad and non-specific bands. Moreover, these bands are not unique to specific biomolecules and are often common between several components of the constructs. For instance, in a previous study, Kandel et al. determined primary peaks associated with collagens and proteoglycans in the range of 2000–2500 nm in dehydrated cartilage, but they could not identify them in the hydrated construct due to the broad and overlapping water band at ∼1900 nm. Moreover, despite identifying minor peaks associated with these macromolecules at ∼1680 and 1720 nm, they were unable to specifically assign these peaks, as they arise from the C–H stretch first overtone vibrations that can reflect the presence of collagen, proteoglycans, or even other organic molecules in the construct, such as the non-neotissue constituents [2,7]. This is a general limitation of analytical NIR spectroscopy, where it is often used as a ‘black-box tool’ without attempting to interpret the chemical information embedded in the spectra [8,40]. Further studies that combine NIR spectroscopy with other analytical methods capable of providing more detailed and specific information about the chemical composition of the sample, such as mass spectrometry, will be necessary to determine the exact macromolecules responsible for the spectral changes involved in the model [41]. However, a variable is considered a confounder if it directly or indirectly affects both the independent and dependent variables regardless of the cause of this effect, and it is not always necessary to determine this cause [42]. Thus, this limitation does not compromise the conclusion of this study; changes in these non-neotissue constituents will be associated with detectable changes in the construct spectra, regardless of the cause of this change, which will compromise the assessment reliability if left uncontrolled. This also highlights the importance of controlling for such confounders, since it will not be possible to determine if the model depends on non-neotissue constituents associated spectral changes.

Identifying and controlling for confounders is essential for the reliability of any study as they can subtly change its results. By demonstrating the confounding effect of non-neotissue constituents on the NIR-based assessment of TECs, these constituents need to be accounted for during study design or analysis to ensure the reliability of the assessment and the results of the study using them [28]. The confounding effect can be avoided during study design through restriction, where all variables except the target variable are kept constant between samples [43]. However, models trained with constant non-neotissue constituents may also include spectral change associated with these constituents, rendering such models only suitable for constructs of specific composition, and thus limiting their generalizability. This will also limit the application of NIR spectroscopy for assessing TECs, for instance, in studying the effect of the non-neotissue constituents on the neotissue.

Conversely, training models using the spectra of samples with diverse non-neotissue constituents, while controlling the effect of these constituents, will allow excluding the non-specific spectral features associated with these constituents. Such models will be more reliable and robust as they depend on conserved neotissue-associated spectral features that are not affected by changes in the non-neotissue constituents. These robust models can then be used for the assessment of TECs with diverse non-neotissue constituents or for monitoring the growth of the neotissue regardless of potential changes in non-neotissue constituents during culture. Moreover, controlling for non-neotissue constituents during model training would be more practical and cost-effective as it will allow the use of samples from multiple expensive and lengthy tissue engineering experiments to achieve the necessary large sample size typically required for ML [1,44].

Ultimately, improving the reliability and effectiveness of NIR-based assessment of TECs will enable the development of non-destructive and cost-effective assessment tools that are needed to support the biomanufacturing of TECs and ensure their quality before clinical use. Although this study only addressed NIR spectroscopy, the approach can be extended to studying the effect of non-neotissue constituents on the reliability of other spectroscopy-based methods, including mid-infrared and Raman spectroscopy. As label-free methods, these non-neotissue constituents might similarly compromise the reliability of these methods, and controlling the effect of these constituents might be necessary [3].

## 5 Conclusions

This study demonstrates that ML algorithms can classify NIR spectra of TECs based on the presence of non-neotissue constituents, independent of the maturity of the constructs. As a result, non-neotissue constituents can be considered confounders for NIR-based assessment of TECs. These confounders may introduce bias during the training of ML models, thus reducing their performance in assessing neotissue-related properties of the constructs—such as the maturity of the constructs. Even subtle modifications in the scaffold or changes in the low-concentration growth factors induce spectral changes significant enough to introduce this bias into the ML process. However, controlling for these confounders, such as by propensity score matching, allows avoiding this bias and improves the performance of the models even if they have a strong influence on the sample's spectra, as seen with the cell-lysis buffer. Thus, to train a reliable and robust NIR spectroscopy-based model for the assessment of TECs, potential confounders (which can be any variable other than the developing neotissue) need to be identified and controlled during the training of the model.

## CRediT authorship contribution statement

**Omar Anwar Elkadi:** Writing - original draft, Investigation, Visualization, Conceptualization, Methodology, Software, Formal analysis. **Florencia Abinzano:** Writing - original draft, Investigation, Methodology. **Ervin Nippolainen:** Investigation, Validation, Methodology, Writing - review & editing. **Ona Bach González:** Investigation. **Riccardo Levato:** Supervision, Resources, Methodology, Validation, Writing - review & editing. **Jos Malda:** Supervision, Validation, Resources, Writing - review & editing. **Isaac O. Afara:** Supervision, Funding acquisition, Conceptualization, Methodology, Validation, Writing - review & editing.

## Declaration of generative AI and AI-assisted technologies in the writing process

During the preparation of this work, Elkadi OA used ChatGPT, Perplexity. ai, and American Journal Experts AI language editing service in order to improve the readability and language of the manuscript. After using these tools, the authors reviewed and edited the content as needed and take full responsibility for the content of the publication.

## Declaration of competing interest

The authors declare that they have no known competing financial interests or personal relationships that could have appeared to influence the work reported in this paper.

## Data Availability

The data and codes required to reproduce these findings are available to download from Mendely Data at https://data.mendeley.com/datasets/6fhp27h9xz/1. Data for Non-Neotissue Constituents as Underestimated Confounders in the Assessment of Tissue Engineered Constructs by Near-Infrared Spectroscopy
