# Supplementary material for: Non-neotissue constituents as underestimated confounders in the assessment of tissue engineered constructs by near-infrared spectroscopy
Source: Mater Today Bio. 2023 Nov 28;24:100879. doi: 10.1016/j.mtbio.2023.100879 (PMC10733684; doi:10.1016/j.mtbio.2023.100879)
Supplement: Supplementary Material 2 [file mmc2.pdf]

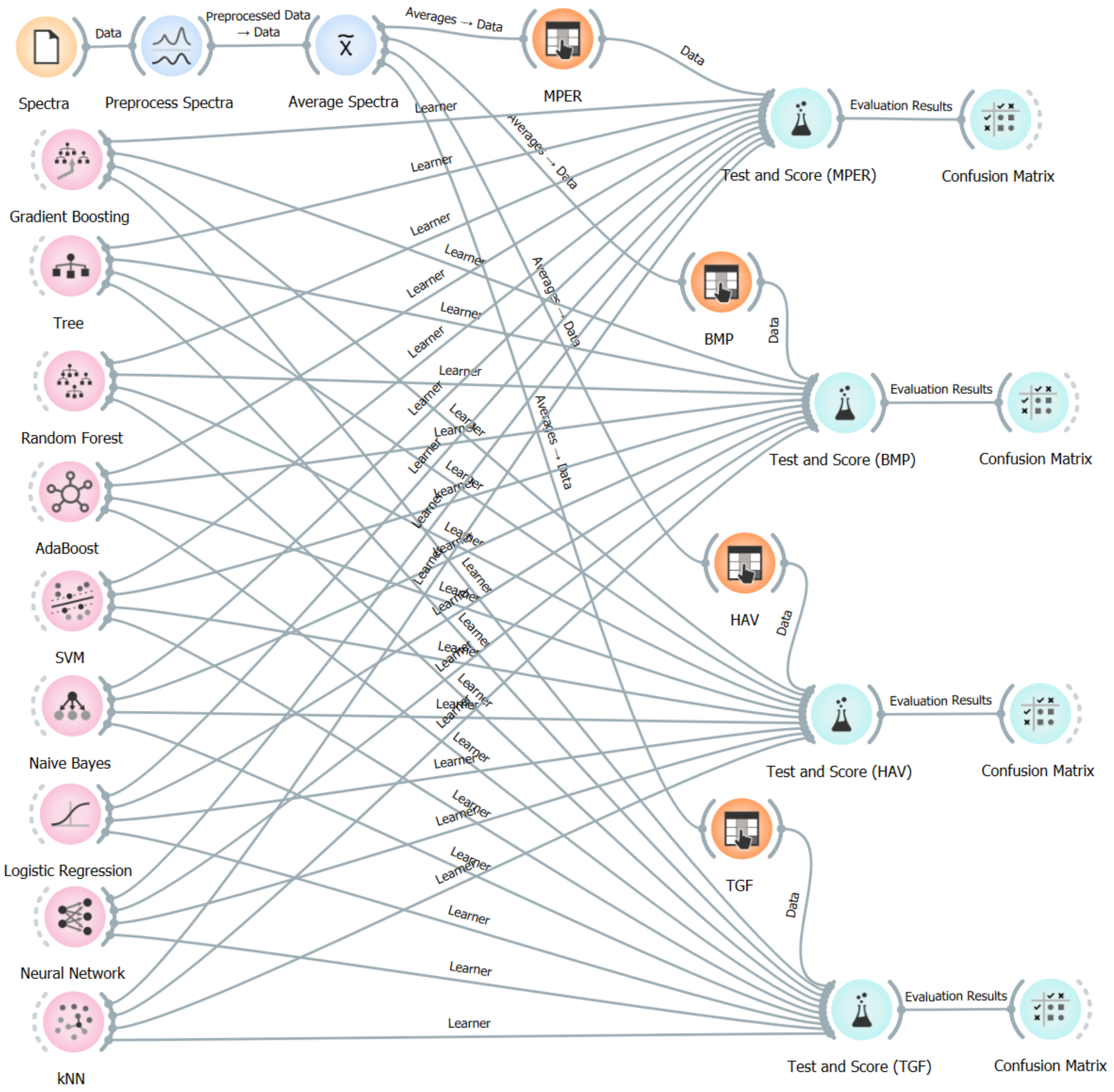

S2.1 The workflow in orange for the evaluation of the performances of the nine machine learning algorithms in classifying the constructs based on the presence of M-PER reagent (MPER), the growth factors BMP-9 (BMP) and TGF-β1 (TGF), and HAV motif (HAV)

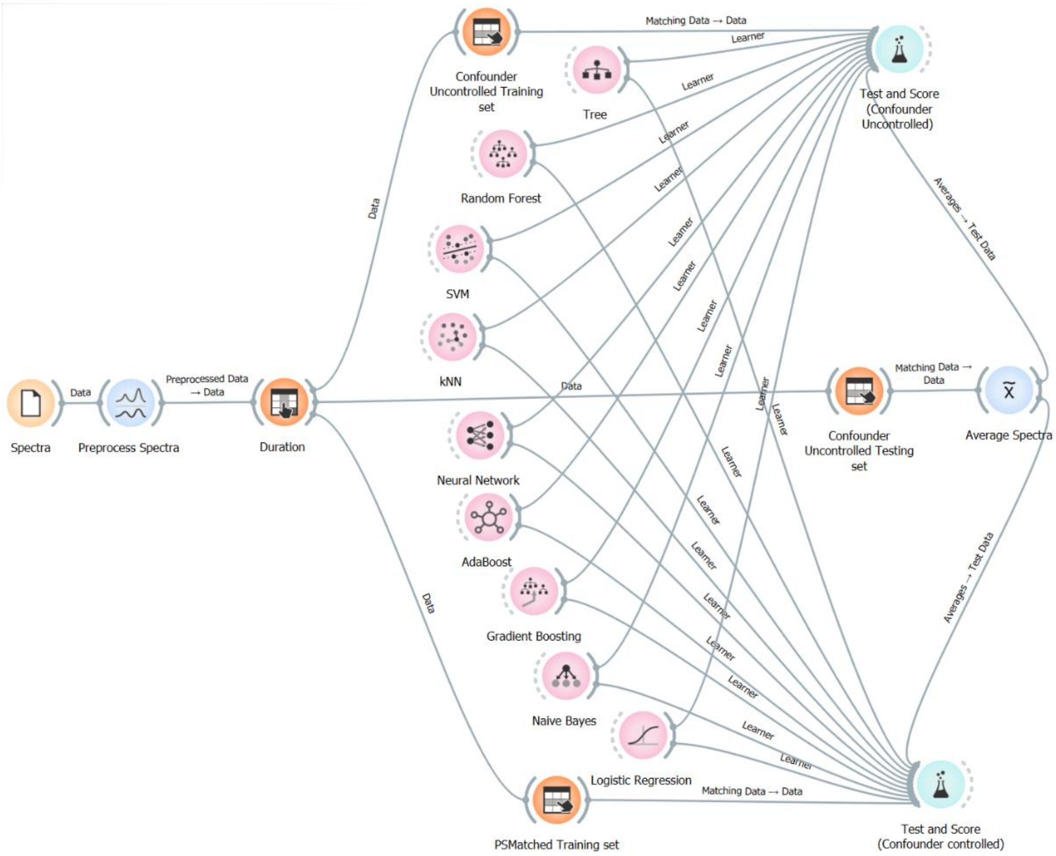

S2.2 The workflow in orange for the evaluation of the performances of 2 sets of nine models in classifying the constructs based on the duration of the incubation (7 days vs 28 days as an indicator of their maturity). One set of these models has been trained using a training set with an uncontrolled confounder (Confounder uncontrolled training set) and the other set has been trained by a training set with all confounders controlled by propensity score matching (PSMatched Training set). Both sets were tested by the same testing sets that include all samples not included in the training of these models. The same workflow has been employed for each of the four confounders (non-neotissue constituents).
